# Psychosexual experiences of men following radiotherapy for prostate cancer in Johannesburg, South Africa

**DOI:** 10.4102/hsag.v23i0.1057

**Published:** 2018-10-09

**Authors:** Matheko N. Phahlamohlaka, Sibusiso Mdletshe, Heather Lawrence

**Affiliations:** 1 Department of Clinical Science, Central University of Technology, South Africa; 2 Department of Medical Imaging and Radiation Sciences, University of Johannesburg, South Africa

## Abstract

**Background:**

Radiation-induced erectile dysfunction (RiED) often occurs among patients diagnosed with prostate cancer (PCa) who undergo radiation treatment. However, sexual health care (SHC) is not a common practice in radiation oncology departments worldwide. Sexual health care in this context refers to a practice of integrating discussions around sexual well-being into the routine follow-up appointments of PCa patients to achieve better patient-centred care. Previous research identified unmet patient needs and mismatched expectations between patients and health care providers regarding SHC, but no such studies have been conducted in a South African setting.

**Aim:**

The aim of this study was to explore and describe the psychosexual experiences of men following radiotherapy for PCa treated in Johannesburg.

**Setting:**

A qualitative phenomenology design with an interpretive research paradigm was employed, which allowed the study objectives to be achieved. Purposive sampling was used to recruit participants from a population of 305 patients. Data were transcribed verbatim and analysed in a step-by-step approach.

**Method:**

Data saturation was reached after completing semi-structured individual interviews (*n* = 9).

**Results:**

Feedback received from the participants was classified into three main themes: (1) their sexual experiences after PCa diagnosis, (2) the impact of losing sexual function on their relationships and (3) the lack of information from medical oncology staff regarding sexual health. Diverse psychosexual experiences and emotional reactions associated with erectile dysfunction before and after radiation therapy were identified.

**Conclusion:**

The loss of sexual function had a detrimental impact on the men’s quality of life, psychological well-being and intimate relationships. Participants shared unsatisfactory feelings about inconsistent and unmet SHC expectations in the radiation oncology clinic.

## Introduction

Prostate cancer (PCa) is a common malignancy among adult men (Rubin & Williams 2001; Washington & Leaver 2016). The 2012 Globocan data show that the incidence rates of PCa are highest in Australasia (111.6 per 100 000) and the United States (97.2 per 100 000), findings that have been attributed to well-established screening practices common in developed countries (Le Roux et al. 2015). According to data from the South African National Cancer Registry, the incidence of PCa in South Africa was 29.4 per 100 000 in 2007, increasing to 67.9 per 100 000 in 2012 (Babb et al. 2014).

Various treatment options are available for localised PCa, including active surveillance, radiotherapy (RT), radical prostatectomy (RP), brachytherapy (BT), cryotherapy (CT) and androgen deprivation therapy (ADT) (Bruce et al. 2012; Challapalli et al. 2012). Radiotherapy is delivered either externally (teletherapy), internally (BT) or concurrently with other modes of treatment (Washington & Leaver 2010). Brachytherapy can also be prescribed as a monotherapy for localised PCa (Skowronek 2013). In addition, RP and ADT are often neoadjuvant and/or adjuvant treatment options for PCa. These modalities are associated with treatment-related erectile dysfunctions in men (Isbarn et al. 2009).

Despite erectile dysfunction (ED) being readily reported in this group of cancer patients (Faiena, Patel & Seftel 2014), several studies have described unmet patient needs and mismatched communication expectations between cancer patients and oncology staff about sexuality and intimacy (Gilbert, Perz & Ussher 2016; Hordern & Street 2007). Counselling aimed at addressing sexual dysfunction is not a common practice in most RT departments (Oskay, Can & Basgol 2014), which could explain the high incidence of psychosocial distress following external beam radiotherapy (EBRT) resulting in sexual dysfunction and decreased psychological well-being experienced by PCa patients (Sharpley, Bitsika & Christie 2008).

Reduced sexual satisfaction, a loss of sexual confidence and a reduction in the ability to respond to sexual stimuli as a secondary psychological consequence of PCa RT have been reported. This is complicated by a reluctance among men to discuss their experiences because embarrassment and feelings of worthlessness often are associated with erectile dysfunction (Badr & Taylor 2009; Perelman & Grill 2013; Wittmann et al. 2009).

Sexual behaviour and attitudes towards sexuality still remain a topic not openly discussed. Male potency and virility are associated with cultural, religious and psychological factors, and a man’s ability to obtain and sustain an erection is considered an important part of the male identity (Perelman & Grill 2013). The taboo nature of discussing male sexuality in African culture was recently addressed at the Wits (University of the Witwatersrand) Institute for Social and Economic Research. The Institute hosted the first conference of the International Association for the Study of Sexuality, Culture and Society (IASSCS) in Africa, in an attempt to demystify African perceptions of human sexuality. The conference was aptly named ‘Sex and Secrecy’ (Amanze 2010; Reid & Walker 2005).

Therefore, it is not surprising that South African health care providers (HCPs) seldom conduct adequate research on sexual health because of the abundance of cultural stereotypes about sexuality within the African mindset (Hall et al. 2012). A stereotypical assumption that men are ready, willing and able to engage in sexual activity at any time or place has contributed to unrealistic expectations regarding sexual functioning in men, and infertile men in particular. African cultures are considered static rather than dynamic and, therefore, it would be very difficult to demythologise the deep-rooted cultural stereotyping and assumptions among Africans. Different cultures perceive sex education differently because of differences in attitudes and beliefs, leading to significant diversity in the management of sex education among the numerous cultural societies across the world (Almahbobi 2012).

Limited information with regard to research on the sexual health of cancer patients is available in the South African health care literature. Unless the experiences of these patients have been explored and described, appropriate guidelines to facilitate sexual counselling and maintain good rapport with patients cannot be developed. Hence, the study aimed to explore and describe the experiences of men following RT for PCa in a South African setting.

## Methods

A qualitative phenomenology design was used, enabling the researcher to explore and describe participants’ lived experiences (Brink, Van der Walt & Van Rensburg 2005) of radiation treatment for PCa. The philosophical groundwork of the study was underpinned in the interpretive paradigm, which supports the view of many truths and multiple realities (Michel 2008). The appropriate choice of a factual paradigm enabled the researcher to answer the research questions from different perspectives, rather than from a distinct perspective.

The study population included approximately 105 PCa patients treated with RT at the research setting each year. The estimated target population of the study included 305 PCa information-rich participants treated between 2011 and 2013. A purposive sampling method was used to recruit participants from this population, applying the criteria outlined in Box 1. The information-rich data in this study were provided by participants who had completed radical EBRT in the past 6 to 18 months. The aforesaid time frame is considered ideal to assess late effects of radiation treatment (Jain et al. 2007). Such participants were seen as a priority to participate in the study interviews. A sample of nine information-rich participants was determined by data saturation. The participants were recruited on the day of their follow-up appointment at the RT centre until data saturation was reached.

Box 1 Inclusion and exclusion criteria applied for the selection of participants.

**Inclusion criteria**

- Patients who completed radical pelvic radiation within the past 6 to 18 months.
- Patients who underwent a radical EBRT three-dimensional (3D) treatment plan; total dose prescription of 66–74 gray (Gy) in 33–37 fractions.
- Good follow-up attendance history.
- Walking patients.
- Patients willing to share their sexual experiences with the researcher.

**Exclusion criteria**

- Patients who had undergone penectomy.
- Patients older than 80 years.
- Patients who received palliative treatment.

EBRT, external beam radiotherapy.

Data were collected from participants by employing semi-structured individual interviews between June 2014 and September 2014. These interviews took place at the research setting. The participants were given an opportunity to express themselves in their language of choice. The majority of the participants spoke isiZulu and an interpreter employed for the study translated English questions into isiZulu for those participants.

The interviews were divided into two phases to obtain both demographic data and information on sexual experiences. In the first part of the interview, the researcher collected demographic information about the participants. The second part explored the psychosexual experiences using the following open-ended key question: ‘How has the PCa diagnosis and treatment affected your sexuality?’ (Brink et al. 2005). Specific models were adapted from the literature to probe the participants during the interviews and included the bring up the topic, explanation, telling, timing, education and recording (BETTER) and permission, limited information, specific suggestions, intensive therapy (PLISSIT) models (Southard & Keller 2009).

The interviews were transcribed verbatim in full text and analysed in a step-by-step approach (Creswell 2003). Transcriptions and field notes were organised and prepared for data analysis. The transcripts were read and reread to obtain a general sense of the patients’ information (Lacey & Luff 2007). The voice recordings were also listened to several times in order to get an impression of the whole experience and to familiarise the researcher with the text.

The information on the transcripts was coded, and three themes and supporting coherent categories emerged from the coding process. Themes were generated from the codes, and the meaning of the data was interpreted and compared to recent data in the literature (Creswell & Plano Clark 2011). Verbatim quotes of the participants were added to substantiate the interpretation of the patients’ experiences.

### Ethical considerations

Ethical approval to undertake the research study was granted by the Academic Ethics Committee (AEC) of the University of Johannesburg (AEC61-01-2013) and by the University of Witwatersrand Human Research Ethics Committee (HREC) (M1311100).

## Results and discussion

The following three themes emerged from the data analysis: (1) the sexual experiences of the participants after PCa diagnosis, (2) the impact of losing sexual function on their relationships and (3) the lack of information from medical oncology staff regarding sexual health. The participants reported their psychosexual distress, frustrations and emotional reactions of the aftermath of the PCa RT.

### The sexual experiences of the participants after prostate cancer diagnosis and external beam radiotherapy

The participants shared many stories describing their sexual experiences after diagnosis of PCa and after treatment with EBRT. Most of their stories focused on distress caused by a deterioration in the ability to achieve an erection, which caused them to experience feelings of sexual inadequacy. The participants reported erectile dysfunction as the most common sexual dysfunction experienced.

‘I was suspecting but immediately after I have been diagnosed then, eh… eh, I became very weak.’ (PSA008, standard 8, pensioner, 71 years)

‘Eh… eh… eh, lack of erection I would say weak erection or unable to engage on sexual activities started while I was getting the treatment with… or radiation therapy.’ (PSA001, no formal education, pensioner, 69 years)

‘I had an erection in the morning. Ja, I used to call it morning glory.’ (PSA003, unknown, unemployed, 62 years)

‘They mostly started after I got injection long time ago. But I was getting a little bit of erection. I think the problem got worse when I started treatment.’ (PSA004, standard 6, unemployed, 68 years)

‘I sleep a little bit and when I wake up, I wake up then having that erection that thinking that knowing that my performance is not hundred percent performing, eh… eh, like I’m performing poor.’ (PSA007, unemployed, 52 years)

Furthermore, participants described a reduced ability to achieve an erection, resulting in an inability to engage in penetrative sexual intercourse. The following verbatim quotes reflect the statements made by the participants:

‘Just can’t get an erection. Sometimes it’s a… it will come thirty percent/forty-five percent.’ (PSA009, certificate, education, 59 years)

‘Forty percent erection and it… it disappears.’ (PSA002, certificate, carpentry, 62 years)

The participants described experiences that were in line with current literature, as radiation-induced erectile dysfunction (RiED) has been reported as a long-term side effect in more than 85% of PCa patients (Sprenkle & Fisch 2007; Thomas & Neal 2013). Although the precise mechanism for RiED is still unclear, possible causes are vascular damage and damage to smooth muscles and nerves (Coates et al. 2015). To a certain extent, emotional, mental and social factors may also result in altered sexual patterns in these men (Carr 2007).

Physical changes reported in the literature include ejaculatory changes and orgasmic problems, such as decreased ejaculation volume, ejaculatory pain, haematospermia and altered orgasmic sensation (Incrocci 2015). White (2015) found that a reduction or loss of semen production is inevitable and irreversible and, therefore, it is important for HCPs to prepare men for this change in sexual functioning before commencing with pelvic RT.

### The impact of losing sexual function on relationships

The participants reported experiences of sexual and emotional detachment from their spouses. They shared stories that described their feelings of guilt when they were unable to achieve intimacy with their partners that sometimes manifested in feelings of inadequacy and frustration. Participants who attempted to buy libido-enhancing medications at the pharmacy to improve sexual intimacy with their spouses reported that these products were financially costly and experienced their efficacy as insignificant. The verbatim quotes that follow describe the experiences of the participants:

‘Ja. Oh, ja, basically I would say, eh… eh… eh, what, eh… eh, the experiences I’m having currently is that I get jealousy [*inaudible*], I got quickly irritated, short-tempered, let me say, I got short… quickly short-tempered.’ (PSA007, unemployed, 52 years)

‘She wants to stick with me through all this. She is supportive but sometimes she gets moody and then she’ll pass remarks like I hope tonight she’s gonna be lucky, something like that there.’ (PSA009, certificate, education, 59 years)

‘I even bought her this electrical thing… I… I… I couldn’t just deny her to have her own desire and all that. So I understand, I am not a capable man of s… sex since I’ve made my… operations and all that.’ (PSA005, standard 9, engineering, 64 years)

‘It didn’t work. Then I decided let me take a full one, which is twenty milligrams.’ (PSA009, certificate, education, 59 years)

The men’s experiences were similar to previously reported findings in the literature. Couples challenged with an illness or disability, such as cancer, experience changes in communication and intimacy patterns (Beck, Robinson & Carlson 2009). The participants shared stories that indicated their perception that sexual intimacy could only be successful when penetrative sex occurs, hence describing feelings of inadequacy because of the reported ED. Bokhour et al. (2001) reported that men’s sexual health is often focused on ED and should be broadened to include desire, arousal and psychosocial aspects of sexual intimacy.

The current literature supports the use of medical interventions to improve sexual intimacy in men with ED, such as oral phosphodiesterase type 5 (PDE5) inhibitors as first-line medical therapy (Mulhall et al. 2010). Second-line therapies include the use of intracorporal injection therapy, vacuum erection device (VED) and intraurethral alprostadil suppositories. In men with severe ED following PCa treatment that is non-responsive to medical therapies, penile prosthesis implantation may be considered as third-line therapy as an effective treatment option (Chung & Brock 2013). Recently, a team of doctors at Tygerberg Hospital in Cape Town, South Africa, successfully preformed a penile transplant on a 21-year-old patient (Bateman 2015), which may indicate the possibility of penile transplantation as a third-line therapy in PCa patients.

### Lack of sexual information received from medical oncology staff

The stories told by participants described being informed by oncology staff about the complications associated with PCa treatment, but without a discussion about sexual health and the possible complications that could result from the prescribed treatment. Participants also described a lack of continuity of care as they frequently were seen by a different oncologist at each visit. This resulted in participants feeling reluctant to discuss sexual health problems, as they had not been able to develop a relationship with a single oncologist or HCP with whom they felt comfortable enough to discuss such matters. Participants also suggested rushed consultations and busy staff as reasons for not discussing sexual health with oncology staff, as reflected by the following statements:

‘What I am trying to say is that every time I gotta explain from start to another doctor, a total stranger, and then the next time I come it’s someone else, I’ve gotta start again, and it gets embarrassing.’ (PSA009, certificate, education, 59 years)

‘You see I picked up a problem with this. As you know every time you see a different doctor.’ (PSA004, standard 6, unemployed, 68 years)

I said to her I got problems and she said to me, okay, she will make a note of it when I come again. (PSA002, certificate, carpentry, 62 years)

‘… like is difficult for him to have a chance to talk about cancer… about some other matters because there are too many patients and the people working here are very busy specially in the machine.’ (PSA004, standard 6, unemployed, 68 years)

There is trending evidence which indicates that although sexual matters are discussed on follow-up appointments, HCPs may require additional support and training to initiate open communication about psychosocial aspects of sexual issues. Such discussions should extend beyond the mechanics of sex in order to reduce the ‘over-medicalisation’ of these concerns (Dyer & Das Nair 2013:2668). Based on the following verbatim statements, it was apparent that some doctors made judgements about psychosexual communication needs of patients based on their age and gender. This misconception in turn sustains unmet sexual needs. Age- and gender-based presumptions may be linked to the psychosocial anxieties and vulnerabilities that HCPs experience when discussing sex and sexuality with patients of particular ages and different sex orientations (Rose, Ussher & Perz 2017), especially in a diverse sociocultural setting.

‘I said to her I got problems and she said to me, okay, she will make a note of it when I come again.’ (PSA002, certificate, carpentry, 62 years)

‘You see I picked up a problem with this. As you know every time you see a different doctor.’ (PSA004, standard 6, unemployed, 68 years)

‘I mean that’s why I… it’s actually better if you speak to like a man-to-man conversation.’ (PSA002, certificate, carpentry, 62 years)

‘… like is difficult for him to have a chance to talk about sex about some other matters because there are too many patients and the people working here are very busy specially in the machine.’ (PSA004, standard 6, unemployed, 68 years)

Krebs (2006) recommended a comprehensive approach for HCPs when communicating information about PCa treatment choices and potential side effects. This holistic approach has been found to reduce controversy and taboos surrounding PCa management (Sinfield et al. 2008). A routine part of oncology follow-ups in most hospitals is often focused on the prognosis of the disease and the administration of radiation treatment (Incrocci 2006; O’Brien et al. 2011), instead of a holistic person-centred approach that addresses issues of sexuality and intimacy.

## Recommendations

It is evident from the results that PCa patients experience psychosexual distress following RT, which affects the quality of their lives. A dedicated sexual counselling clinic in RT departments is recommended in order to promote a holistic approach to men’s sexual health care (SHC). The availability of psychosexual education to address changes in sexuality, sex therapy and couple therapy as a part of PCa care would reassure patients about possible restoration of intimacy and sexuality. The Radiation Oncology Management needs to arrange workshops to create awareness and educate and train HCPs on how to initiate conversations with cancer patients about SHC and may be advised to do so using the PLISSIT and BETTER models. The authors recommend that further research be conducted to develop a model to facilitate sexual counselling for PCa patients in RT departments in South Africa.

The participants in this study voiced concern about a lack of continuity of care in the RT department. They described a need to discuss their experiences with a HCP in a quiet, private space and would prefer to establish a relationship with a HCP when discussing personal experiences such as sexuality. Thus, it is vital to clarify the key roles of each oncology staff member before establishing a dedicated psychosexual counselling clinic for PCa patients.

Lastly, it is recommended that higher education institutions responsible for the training of oncology staff integrate sexual education into the teaching of patient care modules. The role of each member of the multidisciplinary health care team in psychosexual counselling of the patient should be addressed so that oncology staff has the required knowledge and skills to facilitate psychosexual discussions with patients and their partners.

## Conclusion

This study identified three main themes describing the experiences of men after RT for PCa and losing sexual function as a result of RiED. The loss of sexual function had a detrimental impact on the men’s quality of life, psychological well-being and intimate relationships. Participants were dissatisfied with the experience of inconsistent SHC provided in the radiation oncology clinic.

## References

[CIT0001] Almahbobi G, 2012, ‘Multiculturalism and inconsistency in the perception of sex education in Australian society’, *Australasian Medical Journal* 5(12), 623–626. 10.4066/AMJ.2012.1510 23382765 PMC3561589

[CIT0002] Amanze J.N, 2010, *Demythologizing human sexuality in Africa*, viewed 27 August 2017, from http://inerela.org/2010/07/demythologizing-african-conceptions-of-human-sexuality-a-gateway-to-prevention-and-eradication-of-hiv-and-aids-in-africa-prof-james-n-amanze/

[CIT0003] Babb C., Urban M., Kielkowski D. & Kellett P, 2014, ‘Erratum to “prostate cancer in South Africa: Pathology based national cancer registry data (1986–2006) and mortality rates (1997–2009)”’, *Prostate Cancer* 2014, 391257 10.1155/2014/391257, 10.1155/2014/391257 25547986 PMC4274657

[CIT0004] Badr H. & Taylor C.L, 2009, ‘Sexual dysfunction and spousal communication in couples coping with prostate cancer’, *Psychooncology* 18(7), 735–746. 10.1002/pon.1449 19061199 PMC4476400

[CIT0005] Bateman C, 2015, ‘World’s first successful penis transplant at Tygerberg Hospital’, *South African Medical Journal* 105(4), 251–252. 10.7196/SAMJ.9602 26294859

[CIT0006] Beck A.M., Robinson J.W. & Carlson L.E, 2009, ‘Sexual intimacy in heterosexual couples after prostate cancer treatment: What we know and what we still need to learn’, *Urologic Oncology* 27(2), 137–143. 10.1016/j.urolonc.2007.11.032 18367118

[CIT0007] Bokhour B.G., Clark J.A., Inui T.S., Silliman R.A. & Talcott J.A, 2001, ‘Sexuality after treatment for early prostate cancer: Exploring the meanings of “erectile dysfunction”’, *Journal of General Internal Medicine* 16(10), 649–655. 10.1111/j.1525-1497.2001.00832.x 11679031 PMC1495277

[CIT0008] Brink H., Van der Walt C. & Van Rensburg G, 2005, *Fundamentals of research methodology for health care professionals*, 2nd edn, Juta and Company Ltd, Johannesburg.

[CIT0009] Bruce J.Y., Lang J.M., McNeel D.G. & Liu G, 2012, ‘Current controversies in the management of biochemical failure in prostate cancer’, *Clinical Advances in Hematology and Oncology* 10(11), 716–722. 23271258

[CIT0010] Carr S.V, 2007, ‘Talking about sex to oncologists and about cancer to sexologists’, *Sexologies* 16(4), 267–272. 10.1016/j.sexol.2007.06.007

[CIT0011] Challapalli A., Jones E., Harvey C., Hellawell G.O. & Mangar S.A, 2012, ‘High dose rate prostate brachytherapy: An overview of the rationale, experience and emerging applications in the treatment of prostate cancer’, *British Journal of Radiology* 85(Special issue 1), S18–S27. 10.1259/bjr/15403217 23118099 PMC3746400

[CIT0012] Chung E. & Brock G, 2013, ‘Sexual rehabilitation and cancer survivorship: A state of art review of current literature and management strategies in male sexual dysfunction among prostate cancer survivors’, *Journal of Sexual Medicine* 10(Suppl 1), 102–111. 10.1111/j.1743-6109.2012.03005.x 23387915

[CIT0013] Coates J., Jeyaseelan A.K., Ybarra N., David M., Faria S., Souhami L. et al., 2015, ‘Contrasting analytical and data-driven frameworks for radiogenomic modeling of normal tissue toxicities in prostate cancer’, *Radiotherapy and Oncology* 115(1), 107–113. 10.1016/j.radonc.2015.03.005 25818395

[CIT0014] Creswell J.W, 2003, *Research design: Qualitative, quantitative and mixed methods approaches*, 6th edn, Sage, Thousand Oaks, CA.

[CIT0015] Creswell J.W. & Plano Clark V.L, 2011, *Designing and conducting mixed methods research*, 2nd edn, Sage, Thousand Oaks, CA.

[CIT0016] Dyer K. & Da Nair R, 2013, ‘Why don’t healthcare professionals talk about sex? A systematic review of recent qualitative studies conducted in the United Kingdom’, *Journal of Sex Medicine* 10(11), 2658–2670. 10.1111/j.1743-6109.2012.02856.x 22846467

[CIT0017] Faiena I., Patel N. & Seftel A.D, 2014, ‘Prevention of erectile dysfunction after radiotherapy for prostate cancer’, *Asian Journal of Andrology* 16(6), 805–806. 10.4103/1008-682X.133327 25130584 PMC4236318

[CIT0018] Gilbert E., Perz J. & Ussher J, 2016, ‘Talking about sex with health professionals: The experience of people with cancer and their partners’, *European Journal of Cancer Care* 25(2), 280–293. 10.1111/ecc.12216 25040442

[CIT0019] Hall N.M., Morales D.A., Coyne-Beasley T. & St. Lawrence J, 2012, ‘Correlates of African American men’s sexual schemas’, *Sex Roles* 67(11–12), 670–681. 10.1007/s11199-012-0217-4 24031118 PMC3767847

[CIT0020] Hordern A.J. & Street A.F, 2007, ‘Constructions of sexuality and intimacy after cancer: Patient and health professional perspectives’, *Social Science and Medicine* 64(8), 1704–1718. 10.1016/j.socscimed.2006.12.012 17261346

[CIT0021] Incrocci L, 2006, ‘Erectile dysfunction and radiation therapy for prostate cancer’, *Sexologies* 15(2), 116–120. 10.1016/j.sexol.2006.03.005

[CIT0022] Incrocci L, 2015, ‘Radiotherapy for prostate cancer and sexual health’, *Translational Andrology and Urology* 4(2), 124–130. 26813740 10.3978/j.issn.2223-4683.2014.12.08 PMC4708125

[CIT0023] Isbarn H., Boccon-Gibod L., Carroll P.R., Montorsi F., Schulman C., Smith M.R. et al., 2009, ‘Androgen deprivation therapy for the treatment of prostate cancer: Consider both benefits and risks’, *European Urology* 55(1), 62–75. 10.1016/j.eururo.2008.10.008 18945543 PMC3090670

[CIT0024] Jain V.S., Singh K.K., Shrivastava R., Saumsundaram K.V., Sarje M.B. & Jain S.M, 2007, ‘Radical radiotherapy treatment (EBRT + HDR-ICRT) of carcinoma of the uterine cervix: Outcome in patients treated at a rural center in India’, *Journal of Cancer Research and Therapy* 3(4), 211–217. 10.4103/0973-1482.38996 18270396

[CIT0025] Krebs L, 2006, ‘What should I say? Talking with patients about sexuality issues’, *Clinical Journal of Oncology Nursing* 10(3), 313–315. 10.1188/06.CJON.313-315 16789575

[CIT0026] Lacey A. & Luff D, 2007, *Qualitative data analysis*, viewed 03 January 2018, from https://www.rds-yh.nihr.ac.uk/wp-content/uploads/2013/05/9_Qualitative_Data_Analysis_Revision_2009.pdf

[CIT0027] Le Roux H.A., Urry R.J., Sartorius B. & Aldous C, 2015, ‘Prostate cancer at a regional hospital in South Africa: We are only seeing the tip of the iceberg’, *South African Journal of Surgery* 53(3–4), 57–62. 28240486

[CIT0028] Michel C.M, 2008, ‘Implementing a forensic educational package for registered nurses in two emergency departments in Western Australia’, PhD thesis, Fremantle: University of Notre Dame Australia, viewed 03 January 2018, from http://researchonline.nd.edu.au/theses/28/?utm_source=researchonline.nd.edu.au%2Ftheses%2F28&utm_medium=PDF&utm_campaign=PDFCoverPages.

[CIT0029] Mulhall J.P., Bella A.J., Briganti A., McCullough A. & Brock G, 2010, ‘Erectile function rehabilitation in the radical prostatectomy patient’, *Journal of Sexual Medicine* 7(4 Pt 2), 1687–1698. 10.1111/j.1743-6109.2010.01804.x 20388165

[CIT0030] O’Brien R., Rose P., Campbell C., Weller D., Neal R.D., Wilkinson C. et al., 2011, ‘“I wish I’d told them”: A qualitative study examining the unmet psychosexual needs of prostate cancer patients during follow-up after treatment’, *Patient Education and Counseling* 84(2), 200–207. 10.1016/j.pec.2010.07.006 20702055

[CIT0031] Oskay U., Can G. & Basgol S, 2014, ‘Discussing sexuality with cancer patients: Oncology nurses attitudes and views’, *Asian Pacific Journal of Cancer Prevention* 15(17), 321–326. 10.7314/APJCP.2014.15.17.7321 25227836

[CIT0032] Perelman M.A. & Grill E.A, 2013, ‘The role of sex therapy for male infertility’, in Goldstein M. & Schlegel P.N. (eds.), *Surgical and medical management of male infertility*, pp. 204–218, Cambridge University Press, Cambridge.

[CIT0033] Reid G. & Walker L, 2005, *Men behaving differently: South African men since 1994*, Cape Town, Double Storey Books. 10.1080/13691050410001713215 16864199

[CIT0034] Rose D., Ussher J.M. & Perz J, 2017, ‘Let’s talk about gay sex: Gay and bisexual men’s sexual communication with healthcare professionals after prostate cancer’, *European Journal of Cancer Care* 26(1), e12469 10.1111/ecc.12469 26918877

[CIT0035] Rubin P. & Williams J.P, 2001, *Clinical oncology: A multidisciplinary approach for physicians and students*, 8th edn, W.B. Saunders Company, Philadelphia, PA.

[CIT0036] Southard N.Z. & Keller J, 2009, ‘The importance of assessing sexuality: A patient perspective’, *Clinical Journal of Oncology Nursing* 13(2), 213–217. 10.1188/09.CJON.213-217 19349268

[CIT0037] Sharpley C.F., Bitsika V. & Christie D.H, 2008, ‘Psychological distress among prostate cancer patients: Fact or fiction?’, *Clinical Medicine Insights: Oncology* 2, 563–572. 10.4137/CMO.S955 PMC3161698 21892333

[CIT0038] Sinfield P., Baker R., Agarwal S. & Tarrant C, 2008, ‘Patient-centred care: What are the experiences of prostate cancer patients and their partners?’, *Patient Education and Counseling* 73(1), 91–96. 10.1016/j.pec.2008.05.001 18565717

[CIT0039] Skowronek J, 2013, ‘Brachytherapy in the therapy of prostate cancer – An interesting choice’, *Contemporary Oncology (Pozn) [Współczesna Onkologia]* 17(5), 407–412. 10.5114/wo.2013.38557 PMC3934024 24596528

[CIT0040] Sprenkle P.C. & Fisch H, 2007, ‘Pathologic effects of testosterone deprivation’, *Current Opinion in Urology* 17(6), 424–430. 10.1097/MOU.0b013e3282f0ebef 17921778

[CIT0041] Thomas B.C. & Neal D.E, 2013, ‘Androgen deprivation treatment in prostate cancer’, *British Medical Journal* 346, e8555 10.1136/bmj.e8555 23303885

[CIT0042] Washington C.M. & Leaver D.T, 2010, *Principles and practice of radiation therapy*, 3rd edn, Mosby, St. Louis, MO.

[CIT0043] Washington C.M. & Leaver D.T, 2016, *Principles and practice of radiation therapy*, Mosby, St. Louis, MO.

[CIT0044] White I.D, 2015, ‘Sexual difficulties after pelvic radiotherapy: Improving clinical management’, *Clinical Oncology* 27(11), 647–655. 10.1016/j.clon.2015.06.018 26170122

[CIT0045] Wittmann D., Northouse L., Foley S., Gilbert S., Wood D.P. Jr., Balon R. et al., 2009, ‘The psychosocial aspects of sexual recovery after prostate cancer treatment’, *International Journal of Impotence Research* 21(2), 99–106. 10.1038/ijir.2008.66 19158798

